# Chronic postoperative inguinal pain: prevention is better than ‘cure‘

**DOI:** 10.3389/fsurg.2025.1632219

**Published:** 2025-07-23

**Authors:** John M. Findlay, Matthew Lund, Lucy Miller, Jan Vollert, Sarah E. Lamb

**Affiliations:** ^1^ Academic Department of Abdominal Wall and Upper Gastrointestinal Surgery, North Devon District Hospital, Royal Devon University Healthcare NHS Foundation Trust, Barnstaple, United Kingdom; ^2^ NIHR Exeter Biomedical Research Centre, University of Exeter Medical School. St Luke's Campus, Exeter, United Kingdom; ^3^ Department of Clinical and Biomedical Sciences, Faculty of Health and Life Sciences, University of Exeter, Exeter, United Kingdom; ^4^ Department of Pain Management, North Devon District Hospital, Royal Devon University Healthcare NHS Foundation Trust, Barnstaple, United Kingdom; ^5^ Public Health and Sports Sciences, University of Exeter Medical School, St Luke’s Campus, Exeter, United Kingdom

**Keywords:** pain, hernia (inguinal), outcome, prevention, complication

## Introduction

Chronic post-operative inguinal pain (CPIP) is the most common complication of one of the most common operations: inguinal hernia repair. 27% of men, and 3% of women undergo inguinal hernia repair, with over 75,000 repairs performed in the UK per year, and more than 15,000,000 globally (1, 2). Surgery is the only way to definitively treat hernias, and is frequently undertaken to treat pain; however, 20% of patients experience a new or persistent pain 3 months after surgery and beyond, with 10% overall experiencing moderate to severe pain (3, 4).

We do not believe CPIP receives the focus its impact and incidence deserve, both in terms of prevention in clinical practice, and investigation in research. In clinical practice, throughout the evolution and history of inguinal hernia surgery, from the first known attempts by Ancient Roman physician Celsus (or possibly the Ancient Greek physician and anatomist Herophilus), laparoplasty and sclerotherapy by Greensville Dowell and subsequently autoplasty (tissue repair) by Eduardo Bassini in the 19th century, culminating with alloplasty (implant repair) pioneered by Parviz Amid and Irving Lichtenstein which underpins much of modern hernia surgery, long-term repair of the hernia without a significant surgical complication has been always been the primary goal of surgery.

This is mirrored in the outcomes described in a 2015 systematic review of outcome reporting of randomized controlled trials and meta-analyses in groin hernia surgery: 100% reported on recurrence, 80% on traditional complications (such as surgical site occurrence), 50% on generic or hernia-specific quality of life scores, yet only 10% reported on CPIP (5). Furthermore, despite a number of national and international prospective registries reporting on hernia surgery for decades, patient reported outcome measures, particularly pain, are not routinely collected (although pain and other patient reported outcomes are the core outcomes of the recently launched British Hernia Society Registry). However, the prevalence of these outcome measures is inversely proportional to their incidence after surgery; recurrence is experienced by just 2%, traditional complications by another few percent, whilst CPIP is experienced by ten times as many patients (6).

## Why CPIP matters

CPIP matters because of this scale, and its impact on individual patients, as evidenced by the increasing importance of pain within inguinal hernia-specific PROMS. CPIP can be debilitating, exerting profound effects on all aspects of quality of life, patients' ability to work and function, and with further effects on friends, family, society and healthcare providers. Its causes are often complex and overlapping, typically beginning with peripheral factors such as anatomical abnormalities, inflammation, and neuropathy. These can be amplified and sustained by both local and central sensitisation, and further worsened by protective muscle guarding, patient apprehension, and other related conditions. These issues often co-exist and may span multiple disciplines—including surgery, pain management, and musculoskeletal physiotherapy—making effective treatment challenging and resource intensive.

## How we can manage CPIP

The situation is further complicated by the fact that patients often face significant delays in accessing specialist care, which typically falls outside the scope of standard outpatient services. There are few therapies that can be instituted in primary care, and those that can be often cause significant side effects whilst not relieving pain; patients then wait with their symptoms, and this delay risks missing an opportunity to intervene early when the pain is mainly peripherally driven, rather than centrally. This is further complicated by a lack of consensus as to how CPIP should be investigated, assessed and managed, due to a dearth of high-quality evidence. Multiple and often simultaneous or graded interventions may be required, including analgesics (simple, topical, and centrally acting anti-neuropathic agents), percutaneous interventions (such as local anaesthetic and steroid nerve or tender point injections, neuro-ablative or stimulatory therapies), surgical procedures (such as isolated or triple neurectomy, with or without mesh explantation), targeted and general physical therapy, and cognitive therapies. These may take considerable time, confer inconvenience, side effects and risk, and what evidence there is consistently finds relatively low rates of improvement and effect sizes (7, 8).

This may well be due to the difficulties in benchmarking, comparing and recommending treatments when CPIP is considered a coherent syndrome, when in fact it represents an often incoherent mixture of pain mechanisms requiring individual treatment. Those guidelines that exist are often broad, and typically written from a surgical perspective. We are fortunate to offer a dedicated, one-stop, multidisciplinary clinic which offers diagnosis, phenotyping and management, and believe strongly that a multi-speciality synchronic approach in concert is far better rather than sequential and isolated referrals (9). However, despite this it remains extremely difficult for patients, clinicians and healthcare systems to manage.

## How we can prevent it

For these reasons, prevention of CPIP is far preferable to largely unsuccessful attempts to cure it. Indeed the impact and importance of chronic pain after surgery is such that it was identified as one of ten patient-focussed perioperative research priorities by the UK's James Lind Alliance (10). Pre-operative risk factors have been identified, such as younger age, smaller hernias and pre-operative pain and psychological factors, which whilst not making CPIP predictable, does help risk stratification and might guide personalised operations (11). However, a number of studies, meta-analyses and guidelines have identified peri-operative and technical factors identified with CPIP, some consistently and some less so. These include factors which as surgeons we can influence, select or avoid, and are recommended by international guidance for all patients.

For example, it is recommended that we consider mesh implant characteristics (using lower weight mesh for open repair, but not minimally invasive). It is also recommended that we move from routine mesh fixation in minimally invasive approaches to selective fixation (when there is concern that the mesh may migrate), and that if fixation is required we avoid penetrating mesh fixation techniques (for example by using adhesives), or indeed consider a self-gripping mesh. The latter can also be used to avoid penetrating sutures during open repair. It is also now recommended we favour a minimally invasive if possible for all patients undergoing primary repair, and can consider a prophylactic or pragmatic neurectomy, particularly in patients in whom nerves have been disturbed and risk involvement in inflammation and contact with any mesh (12).

Interestingly, surgical experience and/or dedication to hernia surgery is strongly associated with CPIP within RCTs, with rates of 39% in non-experts/specialists and 18% in experts (13), whilst the length of time spent consulting with patients influences their acceptance of the risk of CPIP (in contrast to other “typical” complications) (14). How much this relates to skill, knowledge (and avoidance) of risk factors or perception of the importance of CPIP is unclear, but this—and the fact that compliance with relevant international guidelines is unclear and incomplete (15)—suggest there is more we can do as a community to prevent CPIP. This is not just adherence to specific recommendations; rather also being meticulous in all aspects of surgery to avoid unnecessary inflammation, and exposure and injury of nerves.

By contrast, recurrence and other complication rates have been driven down to just 2% and 3% respectively (2) in these RCTs (indeed it is telling that CPIP is normally not considered within this as a “true” complication'). This has been achieved by the focus of surgeons and researchers on these endpoints, along with other marginal differences in operative time, cost and return to function. These rates and focus, however, are in stark contrast to the impact, implications and incidence of CPIP; suggesting we may be overlooking CPIP (and other patient reported outcomes), in preference to more easily measured surgeon and hospital reported outcomes. Whilst core outcome sets in development for trials of inguinal hernia surgery will no doubt recommend reporting CPIP, we believe the primary goal of inguinal hernia surgery should now move beyond these traditional endpoints, to focus on also preventing CPIP, applying the same level of attention, care, educational, clinical and research efforts to CPIP that have successfully minimised recurrence and surgical complications.
